# *Nocardioides astragali* sp. nov., isolated from a nodule of wild *Astragalus chrysopterus* in northwestern China

**DOI:** 10.1007/s10482-018-1020-1

**Published:** 2018-01-25

**Authors:** Lin Xu, Yong Zhang, Chongyang Li, Xiaoqin Wang, Jinrong Liu, Ville-Petri Friman

**Affiliations:** 1 Key Laboratory of Hexi Corridor Resources Utilization, Hexi University, Zhangye, 734000 Gansu People’s Republic of China; 2 Institute of Agricultural and Biological Technology, Hexi University, Zhangye, 734000 Gansu People’s Republic of China; 3 Department of Biology, University of York, York, YO10 5DD UK; 4 State Key Laboratory of Microbial Resources, Institute of Microbiology, Chinese Academy of Sciences, Beijing, 100101 People’s Republic of China; 5 College of Pastoral Agriculture Science and Technology, Lanzhou University, Lanzhou, 730020 People’s Republic of China

**Keywords:** *Astragalus chrysopterus*, *Nocardioides*, Novel species, Symbiosis

## Abstract

A Gram-positive, non-motile, rod-shaped bacterial strain, designated HH06^T^, was isolated from a nodule of *Astragalus chrysopterus* in northwestern China. Phylogenetic analysis of the 16S rRNA gene sequence showed that the strain is closely related to *Nocardioides alpinus* Cr7-14^T^ and *Nocardioides furvisabuli* DSM 18445^T^ with 98.5 and 98.1% similiarity, respectively. Growth was observed at 4–28 °C in R2A medium (optimum at 25 °C), at 10–30 °C in YMA and LB medium (optimum in both at 28 °C) and at pH 5.0–10.0 in R2A medium (optimum at pH 7.0–8.0). The cell wall peptidoglycan was found to contain LL-diaminopimelic acid as the principal diamino acid and MK-8(H4) was identified as the predominant menaquinone. The major polar lipids were identified as phosphatidylinositol, phosphatidylglycerol, diphosphatidylglycerol, phosphatidylcholine, two unidentified glycolipids and two unidentified polar lipids. The major fatty acids were identified as iso-C_16:0_ (32.8%) and C_18:1_ *ω*9*c* (15.1%). The DNA G+C content of strain HH06^T^ was determined to be 71.4 mol%. Based on phenotypic, chemotaxonomic, phylogenetic properties and DNA–DNA relatedness, it is concluded that strain HH06^T^ represents a novel species of the genus *Nocardioides*, for which the name *Nocardioides astragali* sp. nov. is proposed. The type strain is HH06^T^ (= CGMCC 4.7327^T^ = NBRC 112322^T^).

**Electronic supplementary material:**

The online version of this article (10.1007/s10482-018-1020-1) contains supplementary material, which is available to authorized users.

## Introduction

The genus *Nocardioides*, with *Nocardioides albus* as the type strain, was first proposed by Prauser (1976). At present, the genus contains more than 90 species with validly published names (http://www.bacterio.net/nocardioides.html). Strains of this genus have been isolated from various sources such as soils (Sultanpuram et al. 2015; Sun et al. 2014; Lee and Seong 2014; Srinivasan et al. 2014; Liu et al. 2015), plant rhizosphere (Tuo et al. 2015; Xu et al. 2016; Kämpfer et al. 2016; Glaeser et al. 2014), marine and lake environments (Wang et al. 2016; Deng et al. 2015; Fan et al. 2014; Zhang et al. 2014; Cho et al. 2013a, b) as well as from within animals and plants (Lin et al. 2015).

During a study of the diversity of rhizobial endophytes of wild leguminous plants in July 2015, a strain designated HH06^T^ was isolated from a root nodule of *Astragalus chrysopterus*. Based on phylogenetic analysis, strain HH06^T^ shows around 98% 16S rRNA gene sequence similarity to several members of the genus *Nocardioides*. The taxonomic position of this strain is reported in this paper. Polyphasic taxonomic analyses showed that the strain HH06^T^ is distinct from previously described species of *Nocardioides*, and thus, represents a novel species of this genus, for which the name *Nocardioides astragali* sp. nov. is proposed.

## Materials and methods

### Organisms, maintenance and cultural conditions

Strain HH06^T^ was collected from *A. chrysopterus* in Rouge mountain, Zhangye, China (2880 m; 38°25′58″N, 101°15′06″E) and was isolated on YMA agar plates by using the serial dilution method described by Xu et al. (2014). The YMA medium was prepared according to the instructions from the Deutsche Sammlung von Mikroorganismen und Zellkulturen (DSMZ) (http://www.dsm. de/microorganisms/medium). Plates were incubated at 28 °C for 6 days before isolation of single bacterial colonies, one of which was subsequently selected and cryopreserved at − 80 °C as a suspension in TY (DSMZ) medium supplemented with 30% (w/v) glycerol.

*Nocardioides alpinus* Cr7-14^T^ and *Nocardioides furvisabuli* DSM 18445^T^ were obtained from the China General Microbiological Culture Collection Center (CGMCC) and cultured under the same conditions as the reference strains.

### Phylogenetic analysis and molecular studies

The phylogenetic position of the isolate was determined by 16S rRNA gene sequence analyses. The total DNA of the novel isolate and two closely related reference strains of the genus *Nocardioides* (*N. alpinus* Cr7-14^T^ and *N. furvisabuli* DSM 18445^T^) was extracted by using the method by Marmur (1961). Amplification of the 16S rRNA gene was performed with universal primers P1/P6 (Tan et al. 1997) as described previously (Wang et al. 1999). It is possible to obtain genes associated with rhizobial symbiosis from bacteria isolated from nodules. Therefore, we tested the presence of two important symbiosis genes: *nodA* (acyltransferase) and *nifH* (nitrogenase reductase) as described by Xu et al. (2013).

The almost complete 16S rRNA gene sequence of the novel strain was used for calculating relatedness with its phylogenetic neighbours by using the EzTaxon-e server version 2.1 (http://www.ezbiocloud.net/; Yoon et al. 2016). The phylogenetic analyses based on 16S rRNA sequences of the novel and reference strains belonging to the genus *Nocardioides* were performed by using the software package phylowin (Galtier et al. 1996); multiple alignments were performed by using the CLUSTAL X program (version 1.64b) (Thompson et al. 1997). The phylogenetic tree was constructed by using neighbour-joining methods (Fitch 1971; Saitou and Nei 1987) with the Jukes-Cantor parameter calculation model. The robustness of the topology of the phylogenetic trees was evaluated by bootstrap analyses based on 1000 resamplings (Felsenstein 1985).

The G+C content of DNA was measured by using the thermal denaturation method described by Marmur and Doty (1962) with *Escherichia coli* K-12 DNA as a standard. The DNA-DNA relatedness was determined by using the spectrophotometric method of De Ley et al. (1970).

### Morphological, physiological and biochemical analysis

Strain HH06^T^ was cultivated for 6 days at 25 °C on R2A agar for morphological observation by scanning electron microscopy (Quanta 200; FEI). Gram-staining was performed by using a previously published staining method (Smibert 1994) for cells grown on R2A agar at 25 °C. The growth range and optimum were determined in R2A broth after 6 days of incubation at 4, 10, 20, 25, 30, 37, 40 and 45 °C. The pH range and optimum were determined at pH of 3, 4, 5, 5.5, 6, 6.5, 7, 7.5, 8, 8.5, 9, 10 and 11; KH_2_PO_4_/HCl, KH_2_PO_4_/K_2_HPO_4_ and K_2_HPO_4_/NaOH buffer systems were used to maintain the desired pH. Tolerance to NaCl was examined in R2A broth containing 0–5% of NaCl (w/v, at intervals of 0.5%). Physiological and biochemical properties and enzyme activities were tested by using API 20 NE (identification of Gram negative non-Enterobacteriaceae) and API 50 CH (performance of carbohydrate metabolism) kits (bioMérieux) according to the manufacturers’ instructions. Indole production, reactions in the methyl red, Voges-Proskauer tests, hydrolysis of starch, gelatin, Tween 80, activities of catalase, urease, oxidase, reduction of nitrate and nitrite, and hydrogen sulphide production from cysteine were also determined as described by Smibert (1994). The chitinase, lipase, coagulase and amylase activity were tested as described by Cappuccino and Sherman (1998).

### Chemotaxonomic characterisation

Cellular fatty acid profiles were determined for strains grown on R2A agar (Difco) for 48 h at 25 °C. Fatty acid methyl esters were extracted and prepared by following the standard protocol of the Microbial Identification System (Microbial ID; MIDI). Extracts were analysed by using a Hewlett Packard model HP6890 gas chromatograph equipped with a flame-ionization detector, an automatic sampler, an integrator and a computer, as recommended by the manufacturer. To determine the main isoprenoid quinone, which is an essential component of electron transfer system in the plasma membrane of prokaryotes, strain HH06^T^ and the two reference strains were grown on R2A medium for 6 days at 25 °C with shaking (170 rpm). Extraction and menaquinone assay was performed according to the HPLC method described by Zhang et al. (2003) and Komagata and Suzuki (1988). Briefly, the strains were lyophilised and extracted in methanol. Lipoquinones were analysed by using reversed-phase HPLC and a chromatographic column Diamonsil C18 (200 mm × 4.6 mm, i.d. 5 μm), with 300 ml methanol and 700 ml anhydrous ethanol as the mobile phase. The bacterial biomass for the chemotaxonomic characterisation was obtained from 3-day old cultures grown on medium R2A at 25 °C. The isomer type of the cell wall diaminopimelic acid was analysed as described previously (Staneck and Roberts 1974). Polar lipid profiles were analysed by following the method described by Minnikin et al. (1975). Individual phospholipids were identified by using several spray reagents (Embley and Wait 1994) and through co-migration with authentic standards (Sigma).

## Results and discussion

### Phylogenetic analysis and molecular studies

**Fig. 1:**
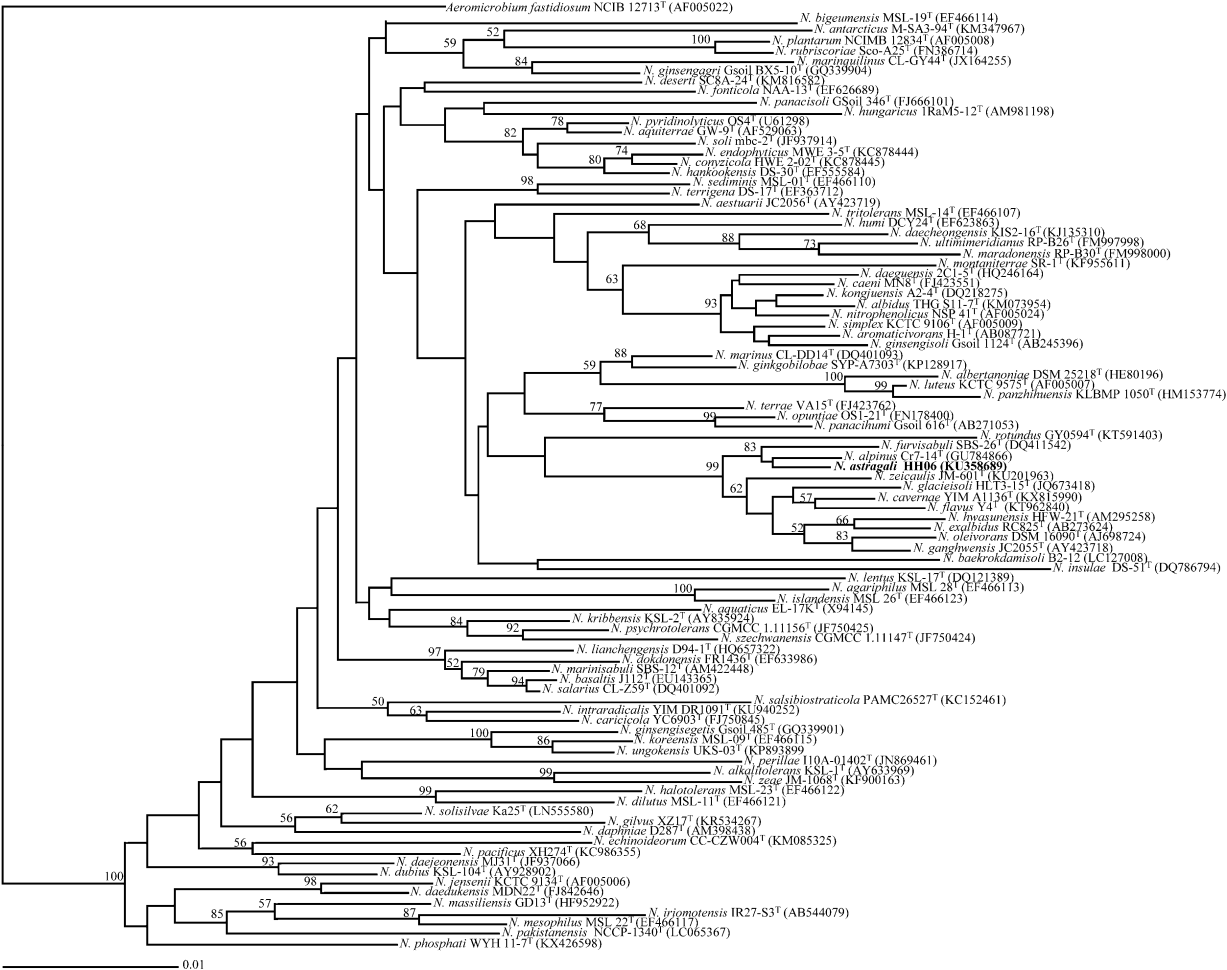
Phylogenetic tree based on 16S rRNA gene sequences of members of the genus *Nocardioides*. The tree was constructed by neighbour-joining method and evolutionary distances were calculated according to the algorithm of Jukes-Cantor model Bootstrap confidence levels (expressed as percentages of 1000 replicates) greater than or equal to 50% are indicated at internodes. GenBank accession numbers are shown in parentheses and the bar denotes for 0.005 nucleotide substitutions per cite

Based on the 16S rRNA gene sequence analysis, strain HH06^T^ is phylogenetically closely related to members of the genus *Nocardioides*. The isolate was found to be closely related to *N. alpinus* Cr7-14^T^ and *N. furvisabuli* DSM 18445^T^ with 98.5 and 98.1% sequence similarities, respectively (Fig. 1), and this was supported by the phylogenetic tree calculated using the maximum parsimony method from Jukes-Cantor distance matrices of the sequences (Supplementary Fig. 1). However, no amplification of *nodA* and *nifH* gene products were observed despite several attempts.

Based on these measurements, the G+C content for the strain HH06^T^ was 71.4 mol% (Table 1), while the DNA-DNA relatedness with the two other reference strains was less than 37 ± 1.2% (SD).

**Table 1:** Distinctive features between the strain HH06^T^ and two closely related reference strains belonging to the genus *Nocardioides*

Characteristic	1	2	3
Growth at 30 °C	**−**	**−**	**+**
Growth at pH6	**+**	**−**	**+**
Growth at 5%NaCl	**−**	**−**	**+**
Hydrolysis of aesculin	**+**	**−**	**−**
Hydrolysis of salicin	**+**	**+**	**−**
Hydrolysis of urea	**+**	**−**	**+**
Hydrolysis of starch	**−**	**−**	**+**
Hydrolysis of gelatin	**+**	**−**	**−**
N-Acetyl glucosamine	**+**	**−**	**+**
Phenylacetic acid	**+**	**−**	**−**
Glycerol	**−**	**−**	**+**
Sucrose	**+**	**−**	**+**
Malate	**+**	**−**	**+**
Citrate	**+**	**−**	**+**
Pyruvic acid sodium	**+**	**−**	**+**
Ribose	**+**	**−**	**+**
l-Arabinose	**+**	**−**	**+**
d-Adonitol	**−**	**−**	**+**
Potassium gluconate	**−**	**+**	**−**
d-Sorbitol	**+**	**−**	**+**
Inositol	**+**	**−**	**+**
Amygdalin	**+**	**−**	**–**
Maltose	**+**	**−**	**+**
Sucrose	**+**	**+**	**−**
ß—Galactosidase	**+**	**−**	**+**
DNA G+C mol%	71.4	71.9	69.1

1. *Nocardioides astragali* HH06^T;^ 2. *N. alpinus* Cr7-14^T;^ 3. *N. furvisabuli* DSM 18445^T^. The symbols denote for negative (−) or positive (+) ability in case of given characteristic. All data were derived from measurements conducted during the present study

### Morphological, physiological and biochemical analysis

Strain HH06^T^ was observed to form tiny convex, smooth, glossy and cream coloured colonies after 6 days growth at 25 °C in R2A medium. Cells were observed to be Gram-positive, rod-shaped (Supplementary Fig. 2) and able to grow between 4 and 28 °C in R2A medium (optimum at 25 °C,) and 10–30 °C in YMA and LB medium (optimum at 28 °C), at pH range of 5.0–10.0 (optimum at pH 7.0–8.0) and at NaCl concentrations lower than 3%. In API tests, strain HH06^T^ was found to be able to utilise d-fructose, gentiobiose, sucrose, malate, citrate, pyruvic acid sodium, ribose, l-arabinose, d-trehalose, d-galactose, d-xylose, d-glucose, d-sorbitol, inositol, mannitol, d-mannose, amygdalin, maltose and sucrose. However, strain HH06^T^ was found to be unable to utilise l-tyrosine, glycerol, asparagine, erythritol, arginine, rhamnose, d-adonitol, xylitol, mannopyranose, d-melezitose, potassium gluconate, l-xylose, l-fucose, d-fucose, sorbose, lactose, d-tagatose or d-melibiose. Additional physiological and biochemical differences between the novel isolate and the two reference strains are provided in the species description and in Table 1.

### Chemotaxonomic characterisation

The main cellular fatty acids of strain HH06^T^ were identified as iso-C_16:0_ (32.8%) and C_18:1_ *ω*9*c* (15.1%) and a full fatty acid profile comparison of strain HH06^T^ and two reference strains is given in Table 2. The predominant menaquinone of strain HH06^T^ was identified as MK-8(H4). Strain HH06^T^ was found to contain LL-diaminopimelic acid as the diagnostic cell wall diamino acid. Phosphatidylinositol, phosphatidylglycerol, diphosphatidylglycerol, phosphatidylcholine, two unidentified glycolipids and two unidentified polar lipids were detected in the polar lipid profile of the strain HH06^T^ (two-dimensional TLCs showed in Supplementary Fig. 3). These chemotaxonomic characteristics are consistent with the classification of most strains in the genus *Nocardioides*.

**Table 2:** The major cellular fatty acids of strain HH06^T^ and two reference strains belonging to the genus *Nocardioides*

Fatty acid	1	2	3
C_14:0_	0.8	0.1	0.2
iso-C14:0	–	1.0	–
C_15:0_	3.5	–	–
C_15:0_ iso	–	1.4	3.0
C_15:0_ anteiso	0.3	0.1	–
C_16:0_	1.3	2.0	2.1
C_16:1_ iso H	3.8	3.5	1.8
C_16:0_ iso	32.8	32.4	29.3
C_17:0_	0.5	3.5	3.3
C_17:0_ iso	5.1	1.1	5.0
C_17:0_ anteiso	1.7	0.3	0.4
C_17:0_ 10-methyl	7.3	1.7	2.1
C_18:0_	0.7	–	0.7
C_18:0_ iso	1.1	0.6	1.1
C_18:0_10-methyl TBSA	3.3	–	–
C_16:0_ 2OH	0.9	–	–
C_17:1_ *ω*8*c*	6.4	39.5	31.2
C_17:1_ *ω*6*c*	2.2	2.3	0.6
C_17:1_ anteiso *ω*9*c*	0.5	–	–
C_18:1_*ω*9*c*	15.1	3.3	10.2
C_18:1_ iso-H	1.1	0.8	0.6
Summed feature 3	2.2	2.5	2.0
Summed feature 6	0.3	0.6	1.6
Summed feature 8	1.8	0.6	1.1
Summed feature 9	7.3	0.8	2.8

1. *Nocardioides astragali* HH06^T^; 2. *Nocardioides alpinus* Cr7-14^T^ (data derived from Zhang et al. 2012); 3. *N. furvisabuli* DSM 18445^T^(data derived from Lee 2007)

Summed Feature 3: C_16:1_ ω7c/C_16:1_ ω6c and/or C_16:1_ ω7c/C_16:1_ ω7c; Summed Feature 6: C_19:1_ ω11c/C_19:1_ ω9c and/or C_19:1_ ω11c/C_19:1_ ω11c; Summed Feature 8: C_18:1_ω7c and/or C_18:1_ω6c; Summed Feature 9: C_17:1_ iso ω9c and/or C_16:0_ 10-methyl

Based on phenotypic, chemotaxonomic, phylogenetic properties and DNA-DNA relatedness, it is concluded that strain HH06^T^ represents a novel species of the genus *Nocardioides*, for which the name *Nocardioides astragali* sp. nov. is proposed. The Digital Protologue database (Rosselló-Móra et al. 2017) TaxoNumber for strain HH06^T^ is TA00381.

### Description of *Nocardioides astragali* sp. nov.

*Nocardioides astragali* (*as.tra’ga.li.* N.L. gen. n *astragali* of *Astragalus* a genus of leguminous plants, referring to the host from which the type strain was isolated).

Cells are Gram-stain positive, short rods, 0.3–0.6 by 0.6–1.1 µm, (occasionally 1.2–2.2 µm in length) after 6 days of growth at 25 °C on R2A agar. Substrate and aerial mycelia are not observed, and colonies on R2A agar are round, convex, glossy with entire margins, cream white, with diameter is 0.1–0.3 cm after 6 days growth at 25 °C. Cells grow better on R2A (optimum at 25 °C) than LB (optimum at 28 °C) or YMA (optimum at 28 °C) media. Growth occurs between 4 and 28 °C in R2A medium, between 10 and 30 °C in YMA and LB media, between pH of 5.0–10.0 (optimum at pH 7.0–8.0) and with NaCl concentrations of 0–3% (w/v). Cells are positive for oxidase, catalase activity, hydrolysis of cellulose, starch, Tweens 80, ß-glucosidase and N-acetyl-β-glucosaminidase and negative for nitrate reduction, urease production and milk peptonisation. The cell wall peptidoglycan contains LL-diaminopimelic acid as the principal diamino acid; MK-8(H4) is the predominant menaquinone. The main cellular fatty acids are iso-C_16:0_ and C_18:1_ *ω*9*c*. Phosphatidylinositol, phosphatidylglycerol, diphosphatidylglycerol and phosphatidylcholine are present as the main polar lipids. The G+C content of the type strain is 71.4 mol%.

The type strain HH06^T^ (= CGMCC 4.7327^T^ = NBRC 112322^T^) was isolated from nodules of *Astragalus chrysopterus* in Zhangye, China. The GenBank accession number for the 16S rRNA gene sequence of strain HH06^T^ is KU358689.

## Electronic supplementary material

Below is the link to the electronic supplementary material.

Supplementary material 1 (PPTX 105 kb)

Supplementary material 2 (TIFF 1202 kb)

Supplementary material 3 (PPTX 303 kb)

Supplementary material 4 (DOCX 24 kb)
