# Supplementary material for: Nocardioides astragali sp. nov., isolated from a nodule of wild Astragalus chrysopterus in northwestern China
Source: Antonie Van Leeuwenhoek. 2018 Jan 25;111(7):1157–63. doi: 10.1007/s10482-018-1020-1 (PMC5999194; doi:10.1007/s10482-018-1020-1)
Supplement: Supplementary file 1 — Supplementary material 1 (PPTX 105 kb) [file 10482_2018_1020_MOESM1_ESM.pptx]

## Slide 1
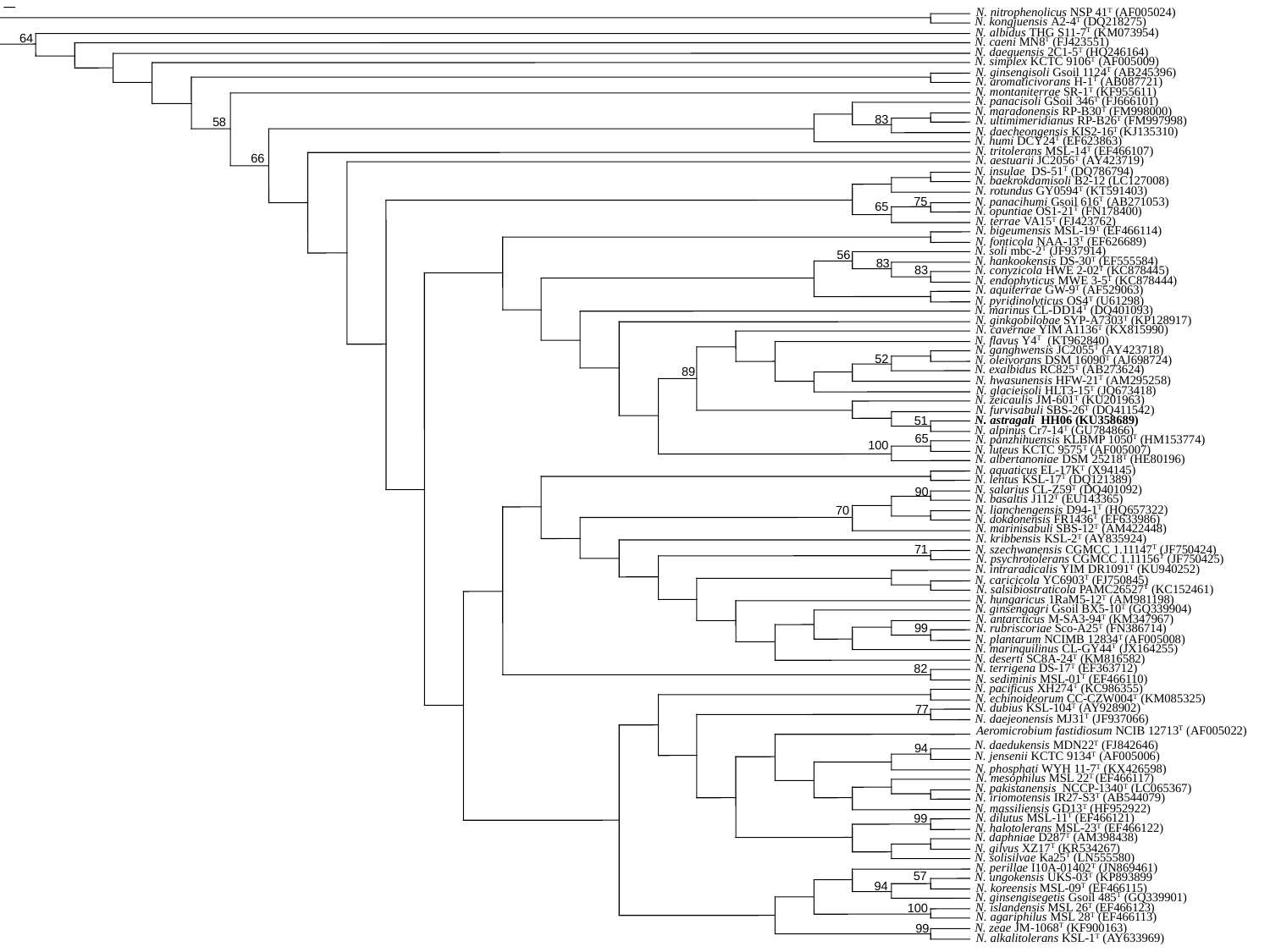

N. nitrophenolicus NSP 41T (AF005024)
N. kongjuensis A2-4T (DQ218275)
N. albidus THG S11-7T (KM073954)
N. caeni MN8T (FJ423551)
N. daeguensis 2C1-5T (HQ246164)
N. simplex KCTC 9106T (AF005009)
N. ginsengisoli Gsoil 1124T (AB245396)
N. aromaticivorans H-1T (AB087721)
N. montaniterrae SR-1T (KF955611)
N. panacisoli GSoil 346T (FJ666101)
N. maradonensis RP-B30T (FM998000)
83
N. ultimimeridianus RP-B26T (FM997998)
N. daecheongensis KIS2-16T (KJ135310)
N. humi DCY24T (EF623863)
N. tritolerans MSL-14T (EF466107)
N. aestuarii JC2056T (AY423719)
N. insulae DS-51T (DQ786794)
N. baekrokdamisoli B2-12 (LC127008)
N. rotundus GY0594T (KT591403)
75
N. panacihumi Gsoil 616T (AB271053)
65
N. opuntiae OS1-21T (FN178400)
N. terrae VA15T (FJ423762)
N. bigeumensis MSL-19T (EF466114)
N. fonticola NAA-13T (EF626689)
N. soli mbc-2T (JF937914)
56
N. hankookensis DS-30T (EF555584)
83
83
N. conyzicola HWE 2-02T (KC878445)
N. endophyticus MWE 3-5T (KC878444)
N. aquiterrae GW-9T (AF529063)
N. pyridinolyticus OS4T (U61298)
N. marinus CL-DD14T (DQ401093)
N. ginkgobilobae SYP-A7303T (KP128917)
N. cavernae YIM A1136T (KX815990)
N. flavus Y4T (KT962840)
N. ganghwensis JC2055T (AY423718)
52
N. oleivorans DSM 16090T (AJ698724)
N. exalbidus RC825T (AB273624)
89
N. hwasunensis HFW-21T (AM295258)
N. glacieisoli HLT3-15T (JQ673418)
N. zeicaulis JM-601T (KU201963)
N. furvisabuli SBS-26T (DQ411542)
N. astragali HH06 (KU358689)
51
N. alpinus Cr7-14T (GU784866)
65
N. panzhihuensis KLBMP 1050T (HM153774)
100
N. luteus KCTC 9575T (AF005007)
N. albertanoniae DSM 25218T (HE80196)
N. aquaticus EL-17KT (X94145)
N. lentus KSL-17T (DQ121389)
N. salarius CL-Z59T (DQ401092)
90
N. basaltis J112T (EU143365)
N. lianchengensis D94-1T (HQ657322)
70
N. dokdonensis FR1436T (EF633986)
N. kribbensis KSL-2T (AY835924)
64
58
66
N. szechwanensis CGMCC 1.11147T (JF750424)
71
N. psychrotolerans CGMCC 1.11156T (JF750425)
N. intraradicalis YIM DR1091T (KU940252)
N. caricicola YC6903T (FJ750845)
N. salsibiostraticola PAMC26527T (KC152461)
N. hungaricus 1RaM5-12T (AM981198)
N. ginsengagri Gsoil BX5-10T (GQ339904)
N. antarcticus M-SA3-94T (KM347967)
99
N. rubriscoriae Sco-A25T (FN386714)
N. plantarum NCIMB 12834T (AF005008)
N. marinquilinus CL-GY44T (JX164255)
N. deserti SC8A-24T (KM816582)
N. terrigena DS-17T (EF363712)
82
N. sediminis MSL-01T (EF466110)
N. pacificus XH274T (KC986355)
N. echinoideorum CC-CZW004T (KM085325)
N. dubius KSL-104T (AY928902)
77
N. daejeonensis MJ31T (JF937066)
Aeromicrobium fastidiosum NCIB 12713T (AF005022)
N. daedukensis MDN22T (FJ842646)
94
N. jensenii KCTC 9134T (AF005006)
N. phosphati WYH 11-7T (KX426598)
N. mesophilus MSL 22T (EF466117)
N. pakistanensis NCCP-1340T (LC065367)
N. iriomotensis IR27-S3T (AB544079)
N. massiliensis GD13T (HF952922)
N. dilutus MSL-11T (EF466121)
99
N. halotolerans MSL-23T (EF466122)
N. daphniae D287T (AM398438)
N. gilvus XZ17T (KR534267)
N. solisilvae Ka25T (LN555580)
N. perillae I10A-01402T (JN869461)
57
N. ungokensis UKS-03T (KP893899
94
N. koreensis MSL-09T (EF466115)
N. ginsengisegetis Gsoil 485T (GQ339901)
N. islandensis MSL 26T (EF466123)
100
N. agariphilus MSL 28T (EF466113)
N. zeae JM-1068T (KF900163)
99
N. alkalitolerans KSL-1T (AY633969)
N. marinisabuli SBS-12T (AM422448)
