# Supplementary figures and images for: Nocardioides astragali sp. nov., isolated from a nodule of wild Astragalus chrysopterus in northwestern China
Source: Antonie Van Leeuwenhoek. 2018 Jan 25;111(7):1157–63. doi: 10.1007/s10482-018-1020-1 (PMC5999194; doi:10.1007/s10482-018-1020-1)

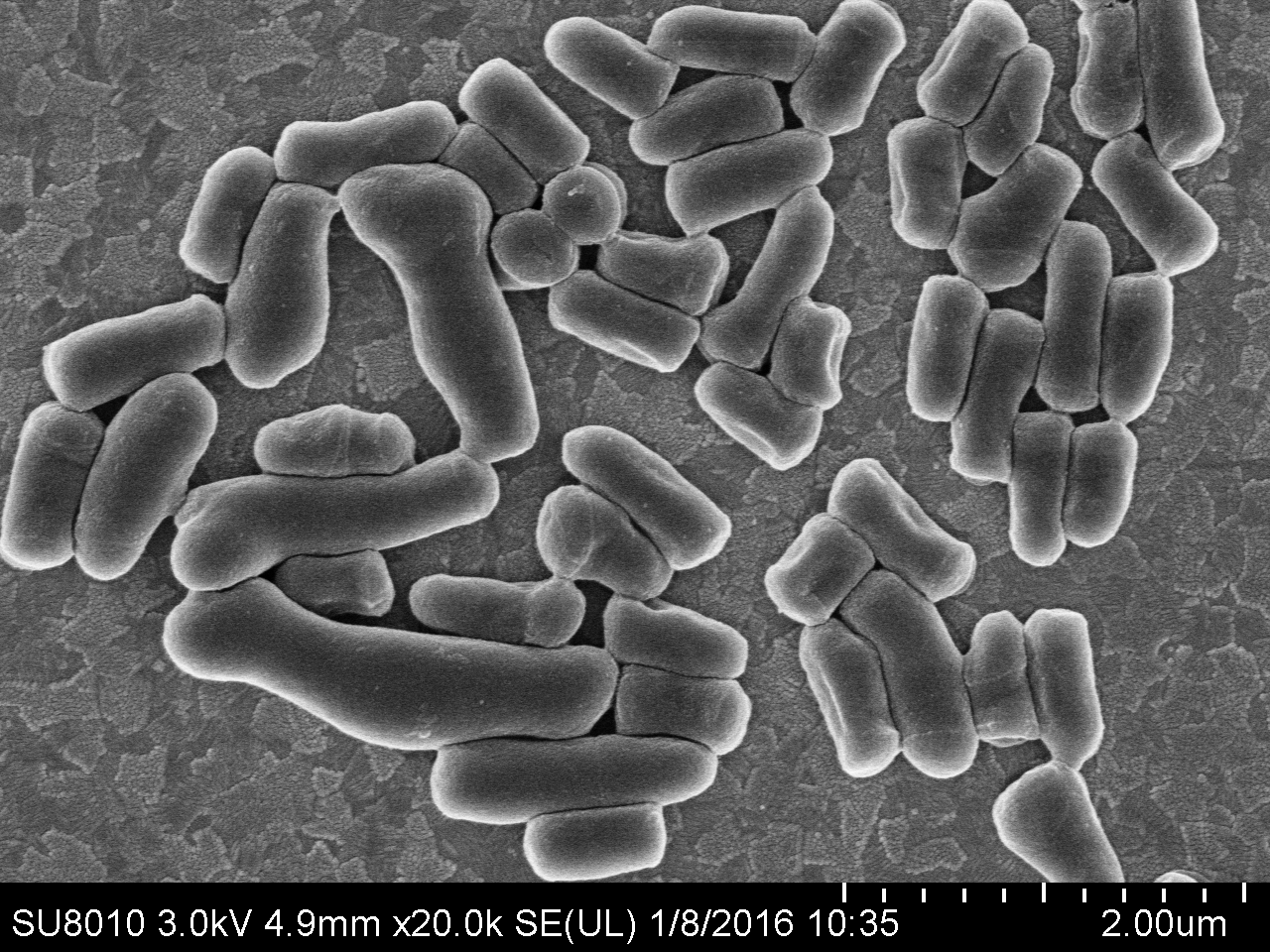

Supplement: Supplementary file 2 — Supplementary material 2 (TIFF 1202 kb) [file 10482_2018_1020_MOESM2_ESM.tif]

## Slide 1
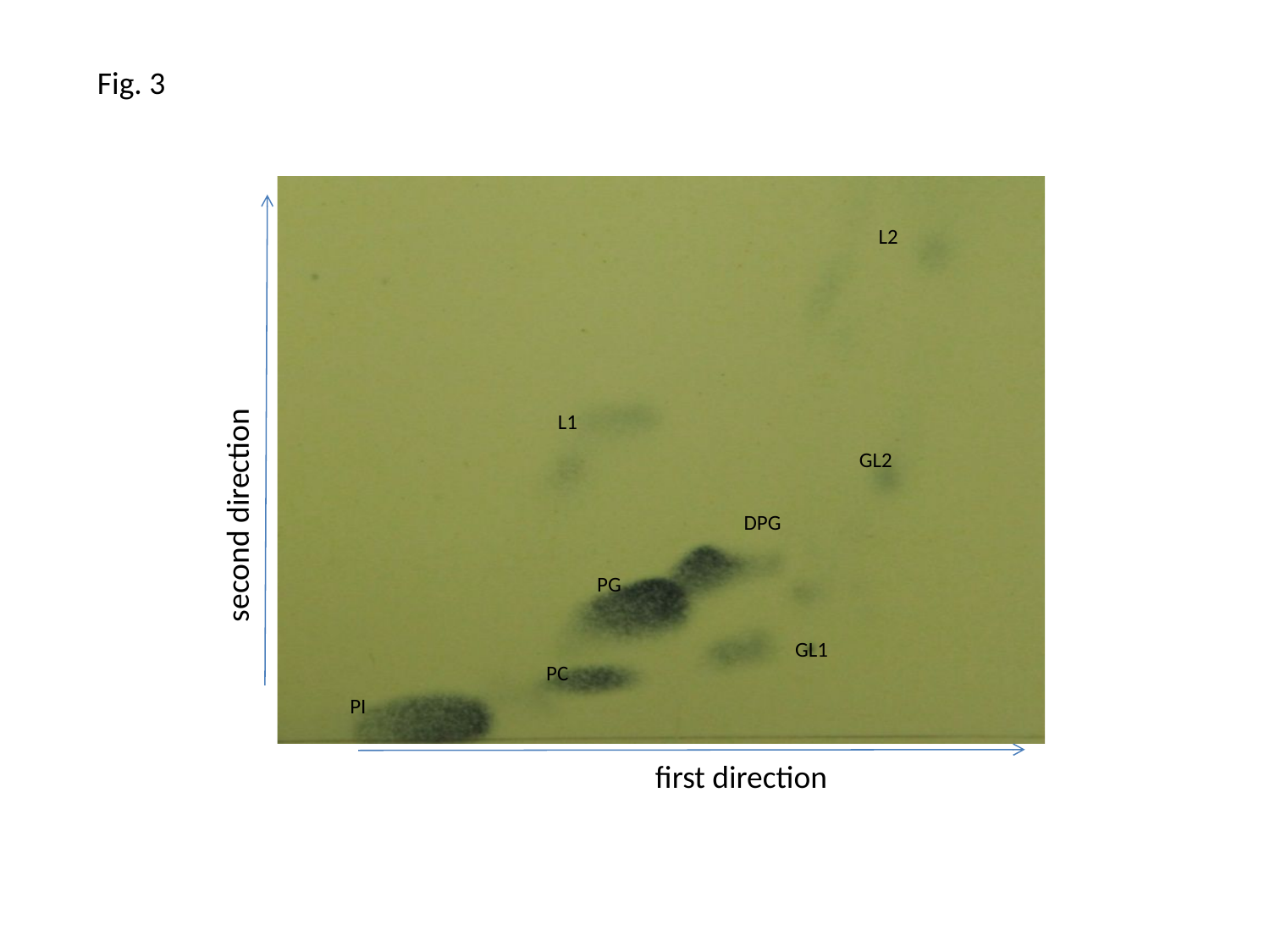

Fig. 3
L2
L1
GL2
DPG
PG
GL1
PC
PI
second direction
first direction

Supplement: Supplementary file 3 — Supplementary material 3 (PPTX 303 kb) [file 10482_2018_1020_MOESM3_ESM.pptx]
