# Supplementary material for: Nocardioides astragali sp. nov., isolated from a nodule of wild Astragalus chrysopterus in northwestern China
Source: Antonie Van Leeuwenhoek. 2018 Jan 25;111(7):1157–63. doi: 10.1007/s10482-018-1020-1 (PMC5999194; doi:10.1007/s10482-018-1020-1)
Supplement: Supplementary file 4 — Supplementary material 4 (DOCX 24 kb) [file 10482_2018_1020_MOESM4_ESM.docx]

**Antonie van Leeuwenhoek Journal of Microbiology**

**Supplementary materials**

***Nocardioides astragali sp.*** ***nov., isolated from a nodule of wild Astragalus chrysopterus in northwestern China***

Lin Xu^a,b,c,d^, Yong Zhang^a,b^, Chongyang Li^e^, Xiaoqin Wang^a^, Jinrong Liu^e^, Ville-Petri Friman^c,*^

*^a^Key Laboratory of Hexi Corridor Resources Utilization, Hexi University, Zhangye 734000, Gansu, PR China*

*^b^Institute of agricultural and biological technology, Hexi University, Zhangye Gansu, 734000, PR China*

*^c^Department of Biology, University of York, York, YO10 5DD, United Kingdom*

*^d^State Key Laboratory of Microbial Resources, Institute of Microbiology, Chinese Academy of Sciences, Beijing, 100101, PR China*

^e^*College of Pastoral Agriculture Scienceand Technology, Lanzhou University, Lanzhou, 730020, PR China*

*Corresponding author: Ville-Petri Friman

Tel: +447411663468

Fax: +447411663468

*Corresponding author: Ville-Petri Friman

Tel: +447411663468 Fax: +447411663468

E-mail address: [ville.friman@york.ac.uk](mailto:ville.friman@york.ac.uk)

Figure S1. Maximum Parsimony tree reconstructed from 16S rRNA gene sequences of the strain HH06^T^ and related reference strains. Bootstrap values (based on 1000 replicates) above 50 % are indicated at the nodes. Bar denotes for 0.1 substitutions per nucleotide position.

Figure S2. Transmission electron micrograph of the strain HH06^T^. Bar indicates 2.0 μm.

Figure S3. Polar lipid profile of the strain HH06T after two-dimensional TLC and staining with molybdatophosphoric acid. PI, phosphatidyl inositol; PG, phosphatidylglycerol; DPG, diphosphatidylglycerol; PC, phosphatidyl choline; GL, unidentified glycolipid; L, unidentified polar lipid.

5
